# Effect of triflumuron, a chitin synthesis inhibitor, on *Aedes aegypti*, *Aedes albopictus* and *Culex quinquefasciatus* under laboratory conditions

**DOI:** 10.1186/1756-3305-6-83

**Published:** 2013-04-04

**Authors:** Thiago Affonso Belinato, Ademir Jesus Martins, José Bento Pereira Lima, Denise Valle

**Affiliations:** 1 Laboratório de Fisiologia e Controle de Artrópodes Vetores, Instituto Oswaldo Cruz-Fiocruz, Rio de Janeiro, RJ, Brazil; 2 Laboratório de Entomologia, Instituto de Biologia do Exército, Rio de Janeiro, RJ, Brazil; 3 Instituto Nacional de Ciência e Tecnologia em Entomologia Molecular, Instituto de Bioquímica Médica, Universidade Federal do Rio de Janeiro, Rio de Janeiro, RJ, Brazil

**Keywords:** Triflumuron, Chitin synthesis inhibitors, *Aedes albopictus*, *Culex quinquefasciatus*, *Aedes aegypti*, Insecticide resistance

## Abstract

**Background:**

Resistance to traditional insecticides represents a threat to the control of disease vectors. The insect growth regulators (IGR) are a potential alternative to control mosquitoes, including resistant populations. The chitin synthesis inhibitors (CSI) are IGRs, which interfere with the insect molting process and represent one major class of compounds against *Aedes aegypti* populations resistant to the larvicide organophosphate temephos. In the present study, we evaluated the efficacy of the CSI triflumuron on *Culex quinquefasciatus*, *Aedes albopictus* and against several *Ae. aegypti* field populations.

**Methods:**

The efficacy of triflumuron, against *Cx. quinquefasciatus* and *Ae. albopictus* was evaluated with laboratory strains through dose–response assays. Additionaly, this CSI was tested against seven *Ae. aegypti* field populations exhibiting distinct resistance levels to both temephos and the pyrethroid deltamethrin. *Aedes aegypti* populations were exposed to both a dose that inhibits 99% of the adult emergence of mosquitoes from the susceptible reference strain, Rockefeller, (EI_99_ = 3.95 μg/L) and the diagnostic dose (DD), corresponding to twice the EI_99_.

**Results:**

Our results indicate that triflumuron was effective in emergence inhibition (EI) of *Cx. quinquefasciatus* (EI_50=_ 5.28 μg/L; EI_90=_ 12.47 μg/L) and *Ae. albopictus* (EI_50=_ 1.59 μg/L; EI_90=_ 2.63 μg/L). Triflumuron was also effective against seven *Ae. aegypti* Brazilian populations resistant to both temephos and deltamethrin. Exposure of all the *Ae. aegypti* populations to the triflumuron EI_99_ of the susceptible reference strain, Rockefeller, resulted in complete inhibition of adult emergence, suggesting no cross-resistance among traditional insecticides and this CSI. However, a positive correlation between temephos resistance and tolerance to triflumuron was observed.

**Conclusion:**

The results suggest that triflumuron represents a potential tool for the control of disease vectors in public health. Nevertheless, they point to the need of constant monitoring of the susceptibility status of vector populations to CSIs.

## Background

*Aedes aegypti*, *Aedes albopictus* and *Culex quinquefasciatus* mosquitoes are widely distributed across the globe, mainly in tropical and subtropical regions [1]. The presence of these species is considered a public health problem, because they are involved in transmission of diseases, such as dengue, yellow fever and lymphatic filariasis [2-4].

In Latin America, the presence of these mosquitoes involves an additional risk, as they are also potential vectors of chikungunya and the West Nile virus, two arboviruses not yet reaching this continent [5-7].

Currently, organophosphates (OP) and pyrethroids (PY) still play an important role in vector control. However, their effectiveness has been hampered due to resistance [8-11]. In this sense, the use of new products is a crucial issue for the development of novel and rational control strategies [12,13].

Compared to conventional insecticides, like OP and PY, insect growth regulators (IGRs) have distinct mechanisms of action and are more selective. Additionally, IGRs are safe for most non-target organisms, and are therefore considered as a promising alternative for insect control [12,14]. Chitin synthesis inhibitors (CSI) are IGRs that interfere with the insect moult. CSIs belong to the benzoyl urea family, a chemical group that has been extensively studied since its discovery in the 1970s [14]. CSI exposure results in deformities of the larval cuticle, which often become unable to survive to the next moult [15-17].

Although the CSI mechanism of action is unclear, there are several reports of its effectiveness against insects, especially in larvae [17-21]. However, since chitin is a molecule present in all life stages of insects, it is likely that a series of mosquito structures are affected as well. Wilson and Cryan, for example, verified that lufenuron affects *Drosophila melanogaster* eggs [22]. Moreover, it has been shown that exposure of larvae to partially lethal doses of CSIs causes a series of disabilities in both viability and reproduction of resulting adults [23-27].

The use of CSIs has been intensified against resistant mosquito populations, given their mechanism of action is distinct from neurotoxic insecticides traditionally employed. Thus, evaluation of the efficacy of CSI on *Ae. aegypti* field populations is essential for rational vector control measures. In addition, the study of CSI effects on other mosquitoes could be important for the development of integrated control strategies.

Under laboratory conditions, triflumuron is effective against *Ae. aegypti*[20]. When larvae are exposed to a sublethal dose, the viability of resulting adults is affected [26]. In the present study, we evaluated the effect of this CSI in several *Ae. aegypti* Brazilian populations with distinct resistance levels to temephos and deltamethrin. Additionally, laboratory strains of *Cx. quinquefasciatus* and *Ae. albopictus*, two other mosquitoes of medical importance, were exposed to several triflumuron doses, and the effects on adult emergence inhibition were evaluated.

## Methods

### Mosquitoes

**Figure 1:**
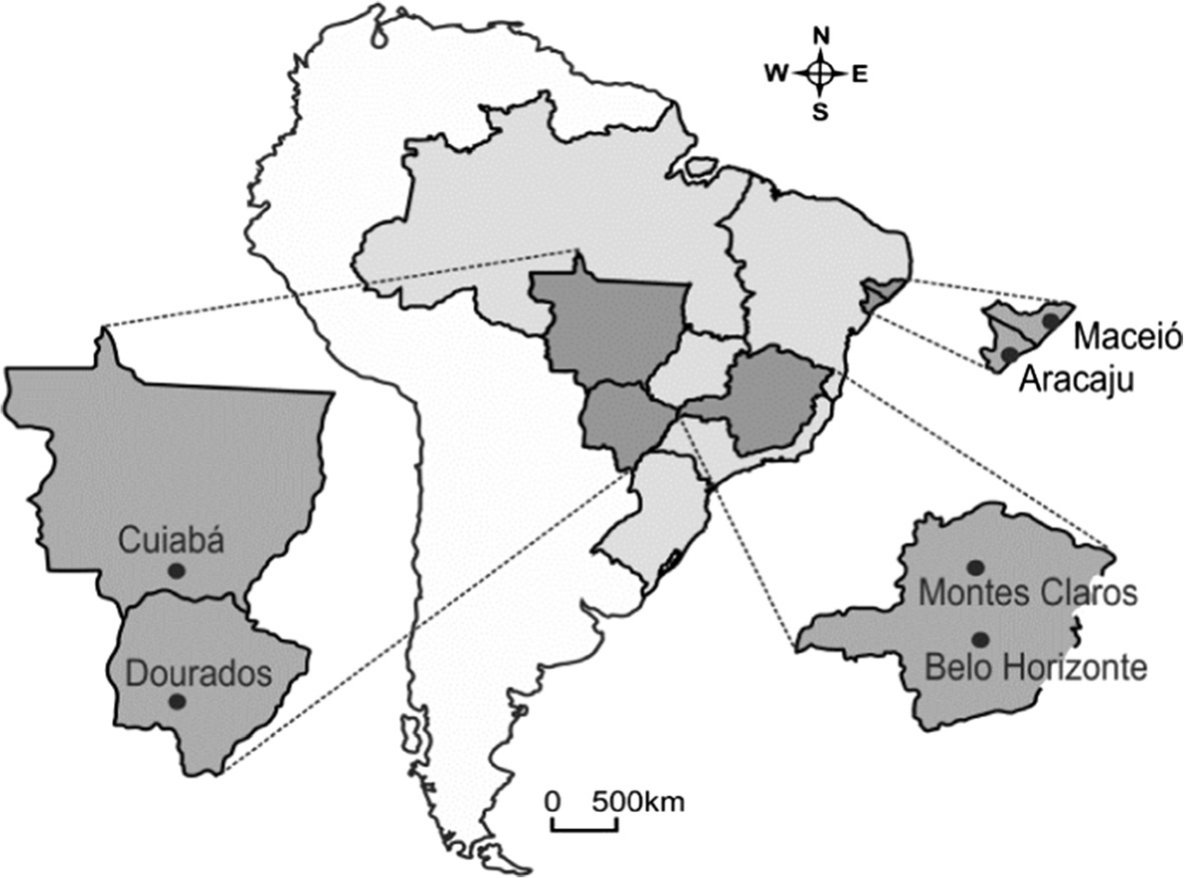
**Geographic location of the *Ae. aegypti *populations adopted in triflumuron assays.**

*Aedes aegypti* populations were chosen according to their geographical location (Figure 1) and resistance levels to both temephos (OP) and deltamethrin (PY). Mosquitoes from the Rockefeller strain, an insecticide-susceptible reference lineage [28], were used as the experimental control.

*Aedes albopictus* and *Cx. quinquefasciatus* strains were collected in Rio de Janeiro city and maintained in the laboratory for five and thirteen years, respectively. However, these laboratory strains are not considered as insecticide susceptible reference strains.

In all cases, groups of 1,000 first instar larvae were reared in plastic basins (33 × 24 × 8 cm) containing 1 L of dechlorinated water and 1 g of cat food (Friskies®, Purina, Camaquã/RS). Larvae from all species and populations were kept in a biological oxygen demand incubator (BOD) at 25 ± 1°C until the third instar, when assays were performed.

### Insecticides

The CSI trifumuron Starycide SC 0.48® (triflumuron 48%) was provided by BayerCropscience. PESTANAL® analytical standard of temephos (97.5%) and deltamethrin (99.7%) were purchased from Sigma-Aldrich.

### Triflumuron bioassays

Dose–response bioassays were performed to evaluate triflumuron efficacy against *Ae. albopictus* and *Cx. quinquefasciatus*. Groups of 10 third instar larvae were exposed to 150 mL of triflumuron solution in transparent plastic cups. The concentrations ranged from 0.25 μg/L to 4.5 μg/L for *Ae. albopictus* and from 2.5 μg/L to 50 μg/L for *Cx. quinquefasciatus*. One milliliter of a 2.5% (w/v) solution of ground cat food (Friskies®, Purina, Camaquã/RS) was supplied once. Eight replicates were used for each concentration. Daily, mortality at each stage was checked and the resulting adults were collected [20,26].

*Aedes aegypti* populations were submitted to two triflumuron doses: one corresponding to the dose inhibiting Rockefeller adult emergence at 99% (EI_99_) and the diagnostic dose (DD), defined as twice the EI_99_[29]. For both concentrations, eight replicates were utilized under the same conditions, as described above. The EI_99_ and DD doses were 3.95 μg/L and 7.9 μg/L, respectively [20]. For all populations, triflumuron assays were performed on the F2 generation; except Maceió (AL), with F3.

In contrast to the neurotoxic insecticides, triflumuron bioassays were monitored for several days, until all insects either died or reached adulthood. Adult emergence inhibition (EI) data were registered as soon as all control group specimens emerged [20]. The results were used to calculate the EI_50_ and EI_90_ values that correspond to the triflumuron doses necessary to inhibit adult development of 50 and 90% of the specimens, respectively.

### *Temephos and deltamethrin bioassays to determine the resistance level of the* Ae. aegypti *populations*

Temephos resistance was evaluated through bioassays [29]. Groups of 20 third instar larvae were exposed to ten temephos concentrations in plastic cups with 100 ml of solution. Four replicates were used for each temephos dose.

Deltamethrin resistance was investigated with adults as described by Da-Cunha *et al.*[9]. Each assay consisted of three 250 mL Wheaton bottles impregnated with the DD of this PY (5 μg/bottle), defined in this assay as the minimal amount of insecticide that kills all the specimens of a susceptible reference strain. One control bottle was impregnated only with acetone as solvent. Each bottle received 20 females, non-blood fed, one to three days old. Knock down (KD) was monitored at 15 min intervals up to 2 hours. Mosquitoes were then transferred to cages free of insecticide and mortality was registered again 24 hours later.

### Statistical analysis

All the assays described here were repeated three times. The trifumuron effective doses EI_50_ and EI_90_ were calculated by probit analysis [30].

Since in Brazil the RR_95_ values are used to guide recommendations of temephos application in the field, the resistance ratios (RR) of *Ae. aegypti* field populations to temephos were calculated with values of the lethal concentrations that kill 95% of larvae (LC_95_). RRs were obtained by dividing the LC of the field populations by the equivalent LC from the Rockefeller strain.

ANOVA was adopted to compare the rate of pupae mortality among *Ae. aegypti* populations. Pearson’s correlation was employed to investigate the relationship between pupae mortality after exposure to triflumuron and resistance levels to temephos of *Ae. aegypti* field populations. The Graph-Pad Prism software version 5.0 for Windows was used to perform these analyzes (GraphPad Software, San Diego California USA, http://www.graphpad.com).

## Results

### *Effect of triflumuron on* Ae. albopictus

**Figure 2:**
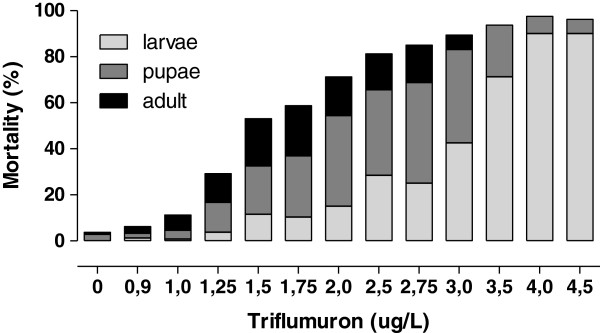
**Stage-specific mortality of *Ae. albopictus *exposed to different triflumuron concentrations.**

The effective EI_50_ and EI_90_ doses inhibiting emergence were 1.59 μg/L and 2.63 μg/L, respectively (Table 1). No adult emergence occurred at concentrations above 3.0 μg/L. Mortality in the control group, not exposed to the CSI, remained below 4%. Figure 2 shows *Ae. albopictus* mortality at each stage and indicates the direct relationship between triflumuron concentration and the precocity of its effects. There were higher larval mortality rates at the highest triflumuron doses. Mortality at the pupal and adult stages was evident mainly in intermediate concentrations, respectively between 1.25 μg/L and 3.5 μg/L and at lower doses, up to 3.0 μg/L.

**Table 1:** Effective doses (ug/L) of triflumuron against the different mosquitoe species evaluated

	Ae. aegypti*	***Ae. albopictus***	***Cx. quinquefasciatus***
**EI50**	**0.86 (0.80-0.93)**	**1.59 (1.54-1.63)**	**5.28 (4.15-6.72)**
**EI90**	**1.80 (1.51-2.17)**	**2.63 (2.42-2.64)**	**12.47 (8.85-17.60)**
**EI99**	**3.95 (2.46-4.49)**	**3.95 (3.35-4.68)**	**25.11 (12.9-48.86)**

Values between parentheses represent the confidence intervals 95% (95% CI).

*Martins *et al.*[20].

### *Effect of triflumuron on* Cx. quinquefasciatus

**Figure 3:**
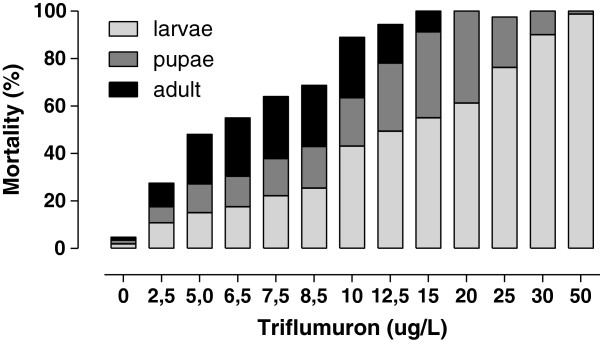
**Stage-specific mortality of *Cx. quinquefasciatus *exposed to different triflumuron concentrations.**

The EI_50_ and EI_90_ values for this species were 5.28 μg/L and 12.47 μg/L, respectively (Table 1). No viable adults were recovered above 15.0 μg/L. Mortality in the control group was 4.7%. Stage-specific mortality of *Cx. quinquefasciatus* exhibited the same pattern observed for *Ae. albopictus* (Figure 3). Larval mortality was directly proportional to the doses employed, while pupal and adult mortality rates were higher at intermediate and lower doses, respectively.

### Effect of triflumuron, deltamethrin and temephos on Ae. aegypti field populations

Effective doses of triflumuron for *Ae. aegypti* remained between 1.5-1.8 fold below the equivalent ones for *Ae. albopictus* (Table 1). Triflumuron was effective against all field populations evaluated, regardeless of their resistance levels to deltamethrin and temephos (Table 2). In all cases, including the Rockefeller strain, mortality in the control groups remained below 3%. Approximately ten days after the initiation of the experiments, all larvae from the control groups, reared without triflumuron, had already reached adulthood. In the same period, up to 86.3% and 99.2% of specimens were dead in the experimental groups exposed to the triflumuron EI_99_ and DD, respectively. Both concentrations completely blocked adult emergence in all populations evaluated (Table 2). However, a small proportion of larvae remained alive for a long period of time. When populations were exposed to EI_99,_ total mortality resulted between 18 (Rockefeller strain) and 24 days (Montes Claros/MG) after the beginning of the bioassays. For DD, total mortality ranged between 17 and 52 days, in Maceió and Montes Claros samples, respectively.

**Table 2:** **Temephos and deltamethrin resistance levels in *Ae. aegypti *field populations**

**Locality/Strain**	**Temephos**	**Deltamethrin**	**Triflumuron,% mort**	
	**RR**_**95**_^**a**^	**% mort DD**^**b**^	**EI**_**99**_	**DD**^**c**^
**Rockefeller**	1.0	100.0	100.0	100.0
**Aracaju/SE**	19.3	88.1	100.0	100.0
**Maceió/AL**	10.3	62.9	100.0	100.0
**Belo Horizonte/MG**	5.4	74.5	100.0	100.0
**Montes Claros/MG**	13.6	62.7	100.0	100.0
**Dourados (North)/MS**	4.3	87.9	100.0	100.0
**Dourados-(South)/MS**	7.1	61.9	100.0	100.0
**Cuiabá/MT**	4.0	89.2	100.0	100.0

^a^In Brazil, localities with *Ae. aegypti *populations exhibiting temephos RR_95 _higher than 3.0 are subjected to insecticide substitution [31]. Note that RR is based on dose–response assays, distinct from the remaining qualitative tests depicted in this table.

^b^the diagnostic dose (DD) of deltamethrin used was 5 μg/bottle [9]. According to this assay, mortality below 80% indicates resistance [32].

^c^the diagnostic dose of triflumuron employed, equivalent to twice the EI_99_, was7.9 μg/L.

**Figure 4:**
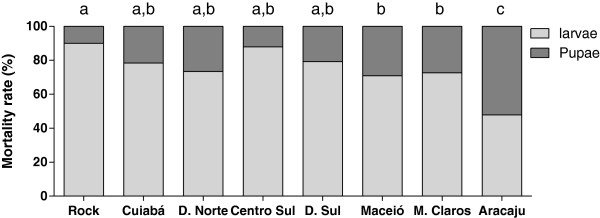
**Stage-specific mortality induced by triflumuron of seven *Ae. aegypti *Brazilian populations exposed to EI**_**99**_**. **Populations are organized according to the resistance level to temephos. Distinct letters above columns indicate significant differences in pupae mortality rates (p < 0.05).

Figure 4 shows the stage-specific mortality of several *Ae. aegypti* populations exposed to triflumuron EI_99_. In this figure, populations are arranged according to their temephos resistance levels, in growth order. The rate of dead pupae in the municipality of Aracaju/SE, the population exhibiting the highest temephos resistance level, was significantly higher than that in other populations (ANOVA; p < 0.05). Significant differences were also evidenced in the rate of pupal mortality of Maceió/AL and Montes Claros/MG when compared to the Rockefeller strain (ANOVA; p < 0.05). Except for Cuiabá/MT and Dourados North/MS, all these populations exhibited high temephos resistance levels, as shown in Table 2 (RR_95_ > 10.0).

In general, when field populations were exposed to triflumuron EI_99_, a positive correlation between temephos resistance levels and the rate of pupal mortality was found (r^2^ = 0,7293; p < 0,05). The same correlation was not observed when populations were exposed to DD. In contrast to temephos resistance evaluation, deltamethrin bioassays were limited to exposure to a single dose of the pyrethroid. According to this bioassay, all *Ae. aegypti* populations were classified as resistant to deltamethrin (Table 2). Nevertheless, there was never a correlation between deltamethrin mortality rates and pupae mortality in *Ae. aegypti* populations exposed to EI_99_ and DD of triflumuron noted.

## Discussion

Insecticide resistance is a growing problem that hampers mosquito control in different regions around the world. Nowadays, resistance to the main neurotoxic insecticide classes has spread among several populations of insect disease vectors. Therefore, biological control methods and the use of chemicals with distinct mechanisms of action have been increasingly employed. The utilization of chitin synthesis inhibitors, for example, represents one major current strategy for mosquito chemical control. For this reason, evaluation of the efficacy of such compounds on mosquito field populations is essential. This work deals with the CSI triflumuron efficacy against *Ae. aegypti* field populations, as well as laboratory colonies of *Ae. albopictus* and *Cx. quinquefasciatus*.

Our results indicate that triflumuron, under laboratory conditions, is effective against the three mosquito species evaluated. As expected, this compound induced adult emergence inhibition of *Ae. albopictus* and *Cx. quinquefasciatus* in a dose-dependent way. Previous results of our group have proven that this compound acts in a similar way on *Ae. aegypti*[20]. Novaluron, another CSI, has the same effect on this mosquito [17]. Moreover, other IGRs, such as methoprene (juvenile hormone mimic) and halofenozide (ecdysone agonist), exhibited similar results [33,34].

Several authors have observed that high doses of CSIs caused more pronounced mortality of mosquito larvae, relative to pupae and adults [17,18,35]. These results corroborate the data presented here, performed with triflumuron, and confirm the direct relationship between CSI concentrations and the precocity of their effects.

Triflumuron EI_50_ and EI_90_ against *Cx. quinquefasciatus* (Table 1) were 3.3 and 4.7 times higher than the corresponding values in *Ae. albopictus*. In this latter species, in turn, triflumuron effective doses were around 1.5 - 1.8 times higher than in *Ae. aegypti* (EI_50_ and EI_90_ of 0.86 μg/L and 1.8 μg/L, respectively) [20]. The triflumuron doses recommended by the manufacturer for *Aedes* and *Culex* control are around 0.12 g/L and 0.24 g/L, respectively [36], values much higher than the effective concentrations for these culicids.

Although there are some reports related to field simulated assays, to our knowledge this is the first evaluation of the triflumuron effect on *Ae. albopictus* under laboratory conditions. With respect to *Cx. quinquefasciatus*, our results slightly differ from other reports, which declared EI_50_ and EI_90_ values of 2.0 and 7.0 μg/L, respectively [37,38]. Intraspecific variations of IGR effective doses among different laboratories are very common and are derived, in part, from the use of distinct protocols, as already discussed by Braga *et al.*[34]. Additionally, variations in effective doses among different CSIs for a given species, or among different insect species for a given CSI, have also been reported. Diflubenzuron, the first chitin synthesis inhibitor commercially available, has been used in the control of various insect species, especially in agriculture [14]. Concentrations of 0.5 μg/L of this compound resulted in 50% of *Cx. quinquefasciatus* larval mortality [37]. Su *et al.* found for novaluron, another promising CSI, EI_50_ and EI_90_ of respectively 0.16 and 0.60 μg/L for *Cx. quinquefasciatus*[35]. Ali *et al.* studied the effect of diflubenzuron in *Ae. albopictus* and encoutered an EI_50_ of 0.45 μg/L for this species, the EI_90_ being 0.84 μg/L [39]. Hexaflumuron, another CSI, also proved very effective against *Ae. albopictus*, with the EI_50_ of 0.2 μg/L [40].

Although effective doses of triflumuron are higher when compared to other IGRs, Mian and Mulla emphasized the efficient activity of this CSI on mosquitoes, which is confirmed by its effectiveness in field trials [14]. Sulaiman *et al.* verified that 14.0 mg/L of triflumuron inhibited *Ae. albopictus* emergence for 168 days under simulated field conditions [41]. Likewise, Batra *et al.* found that triflumuron was effective against *Cx. quinquefasciatus* and *Anopheles stephensi* for seven weeks [42].

We also demonstrated that triflumuron is effective against *Ae. aegypti* field populations exhibiting different resistance levels to organophosphates and pyrethroids. In such cases, a small number of larvae always remained alive for many days, since the primary action of CSIs is not to induce mortality, but to interfere with development.

*Aedes aegypti* larval mortality was more pronounced when populations were treated with DD rather than EI_99_, confirming the direct relationship between CSI concentration and the precocity of its effects, likewise this has previously been shown for the Rockefeller strain [20]. However, total mortality occurred later in populations exposed to DD. Empirical observations during CSI bioassays indicate that in many cases, a small number of larvae remain alive for long periods, regardless of the dosage. However, these larvae never progress to adulthood. Probably, this occurs due to natural variations among specimens of field populations.

The absence of adult emergence when field populations are exposed to triflumuron, repudiates cross-resistance between this CSI and neurotoxic inseticides. However, a positive correlation between pupal mortality rates and the temephos resistance ratios was noted, suggesting a potential triflumuron tolerance in populations resistant to this OP. This triflumuron tolerance could derive from the increased activity of enzymes related to metabolic resistance, already detected in several Brazilian *Ae. aegypti* populations [10,34]. In contrast, correlation between deltamethrin mortality rates and triflumuron induced pupae mortality in these *Ae. aegypti* populations were not detected. This result may in part be associated with the qualitative nature of the PY bioassay, consisting of a single dose. Although there are few reports concerning IGR resistance, diflubenzuron tolerance associated with mixed-function oxidases was evidenced in blowflies [43]. In mosquitoes however, to date IGR resistance has not yet been directly associated with increased activity of detoxifying enzymes.

## Conclusions

Our results indicate that triflumuron was effective against the three mosquito species evaluated. Furthermore, this CSI was also potent against seven *Ae. aegypti* populations resistant to the two major classes of insecticides currently adopted in vector control. IGRs generally offer low toxicity to mammals, including man. Therefore, triflumuron might be considered an alternative for mosquito control in urban areas, although it has not yet been approved for application in drinking water, which precludes its use against *Ae. aegypti*[44].

## Competing interests

The authors declare that they have no competing interests.

## Authors’ contributions

TAB designed the study, carried out all experiments and drafted the manuscript. AJM and DV also participated with the study design and critically reviewed the manuscript. JBPL scrutinized the protocol for the study and contributed with the interpretation of results. All authors read and approved the final version of the manuscript.

We dedicate this paper to the memory of Alexandre A. Peixoto, an outstanding friend and scientist.
